# The OPTIMIZE patient- and family-centered, primary care-based deprescribing intervention for older adults with dementia or mild cognitive impairment and multiple chronic conditions: study protocol for a pragmatic cluster randomized controlled trial

**DOI:** 10.1186/s13063-020-04482-0

**Published:** 2020-06-18

**Authors:** E. A. Bayliss, S. M. Shetterly, M. L. Drace, J. Norton, A. R. Green, E. Reeve, L. A. Weffald, L. Wright, M. L. Maciejewski, O. C. Sheehan, J. L. Wolff, K. S. Gleason, C. Kraus, M. Maiyani, M. Du Vall, C. M. Boyd

**Affiliations:** 1 Institute for Health Research, Kaiser Permanente Colorado, Aurora, CO USA; 2 Department of Family Medicine, University of Colorado School of Medicine, Aurora, CO USA; 3 Division of Geriatric Medicine and Gerontology, Johns Hopkins University School of Medicine, Baltimore, MD USA; 4 Quality Use of Medicines and Pharmacy Research Centre, UniSA: Clinical and Health Sciences, University of South Australia, Adelaide, SA Australia; 5 Geriatric Medicine Research, Faculty of Medicine, and College of Pharmacy, Dalhousie University and Nova Scotia Health Authority, Halifax, NS Canada; 6 Department of Clinical Pharmacy, Kaiser Permanente Colorado, Aurora, CO USA; 7 Durham Center of Innovation to Accelerate Discovery and Practice Transformation (ADAPT), Durham, Veterans Affairs Medical Center, Durham, NC USA; 8 Department of Population Health Sciences, Duke University Medical Center, Durham, NC USA; 9 School of Public Health, Johns Hopkins School of Medicine, Baltimore, MD USA

**Keywords:** Polypharmacy, Multimorbidity, Deprescribing, Dementia

## Abstract

**Background:**

Most individuals with dementia or mild cognitive impairment (MCI) have multiple chronic conditions (MCC). The combination leads to multiple medications and complex medication regimens and is associated with increased risk for significant treatment burden, adverse drug events, cognitive changes, hospitalization, and mortality. Optimizing medications through deprescribing (the process of reducing or stopping the use of inappropriate medications or medications unlikely to be beneficial) may improve outcomes for MCC patients with dementia or MCI.

**Methods:**

With input from patients, family members, and clinicians, we developed and piloted a patient-centered, pragmatic intervention (OPTIMIZE) to educate and activate patients, family members, and primary care clinicians about deprescribing as part of optimal medication management for older adults with dementia or MCI and MCC. The clinic-based intervention targets patients on 5 or more medications, their family members, and their primary care clinicians using a pragmatic, cluster-randomized design at Kaiser Permanente Colorado. The intervention has two components: a patient/ family component focused on education and activation about the potential value of deprescribing, and a clinician component focused on increasing clinician awareness about options and processes for deprescribing. Primary outcomes are total number of chronic medications and total number of potentially inappropriate medications (PIMs). We estimate that approximately 2400 patients across 9 clinics will receive the intervention. A comparable number of patients from 9 other clinics will serve as wait-list controls. We have > 80% power to detect an average decrease of − 0.70 (< 1 medication). Secondary outcomes include the number of PIM starts, dose reductions for selected PIMs (benzodiazepines, opiates, and antipsychotics), rates of adverse drug events (falls, hemorrhagic events, and hypoglycemic events), ability to perform activities of daily living, and skilled nursing facility, hospital, and emergency department admissions.

**Discussion:**

The OPTIMIZE trial will examine whether a primary care-based, patient- and family-centered intervention educating patients, family members, and clinicians about deprescribing reduces numbers of chronic medications and PIMs for older adults with dementia or MCI and MCC.

**Trial registration:**

NCT03984396. Registered on 13 June 2019

## Administrative information

Note: the numbers in curly brackets in this protocol refer to SPIRIT checklist item numbers. The order of the items has been modified to group similar items (see http://www.equator-network.org/reporting-guidelines/spirit-2013-statement-defining-standard-protocol-items-for-clinical-trials/).

**Table:** 

Title {1}	The OPTIMIZE patient- and family-centered, primary care-based deprescribing intervention for older adults with dementia or mild cognitive impairment and multiple chronic conditions: study protocol for a pragmatic cluster randomized controlled trial
Trial registration {2a and 2b}.	Trial Registration: This trial was registered on June 13, 2019, at clinicaltrials.gov (NCT03984396 – retrospectively registered), https://clinicaltrials.gov/ct2/show/NCT03984396
Protocol version {3, 25}	At the time of article submission, the intervention is being delivered to the initial intervention group under protocol Version 5, January 25, 2020. Any protocol modifications are communicated by the study investigators to the KPCO IRB, DSMB, and study team.
Funding {4}	Funder: National Institute on Aging (R33-AG057289)
Author details {5a}	Bayliss EA*^1,2^, Shetterly SM^1^, Drace ML^1^, Norton J^3^, Green AR^3^, Reeve E^4^, Weffald LA^5^, Wright L^1^, Maciejewski ML^6,7^, Sheehan OC^3^, Wolff JL^8^, Gleason KS^1^, Kraus C^1^, Maiyani M^1^, Du Vall M^5^, Boyd CM*^3^ ^1^ Institute for Health Research, Kaiser Permanente Colorado, Aurora, CO ^2^ Department of Family Medicine, University of Colorado School of Medicine, Aurora, CO ^3^ Division of Geriatric Medicine and Gerontology, Johns Hopkins University School of Medicine, Baltimore MD ^4^ Quality Use of Medicines and Pharmacy Research Centre, UniSA: Clinical and Health Sciences, University of South Australia, Adelaide, SA, Australia ^5^ Geriatric Medicine Research, Faculty of Medicine, and College of Pharmacy, Dalhousie University and Nova Scotia Health Authority, Halifax, NS, Canada ^5^ Department of Clinical Pharmacy, Kaiser Permanente Colorado, Aurora, CO ^6^ Durham Center of Innovation to Accelerate Discovery and Practice Transformation (ADAPT), Durham, Veterans Affairs Medical Center, Durham, NC ^7^ Department of Population Health Sciences, Duke University Medical Center, Durham, NC ^8^ School of Public Health, Johns Hopkins School of Medicine, Baltimore, MD * Drs. Bayliss and Boyd are co-Principal Investigators of the OPTIMIZE trial
Name and contact information for the trial sponsor {5b}	National Institute on Aging. www.nia.nih.gov National Institute on Aging Building 31, Room 5C27 31 Center Drive, MSC 2292 Bethesda, MD 20892 1-800-222-2225
Role of sponsor {5c} {21a}	The funder had no role in developing the study design, data collection, management, interpretation or analysis, writing of the manuscript, or decision to submit the manuscript for publication. The funder does not have authority over any of these activities. The funder did contract with members of a Data Safety Monitoring Board.

## Contribution to the literature

- Deprescribing interventions in populations with cognitive impairment have been largely limited to inpatient or skilled nursing settings or focused on specific medication classes. This protocol describes the development of a pragmatic intervention for this population that targets multiple medications with the aim of being integrated and sustained in routine clinical practice.
- This study will test the effectiveness of an intervention that combines patient and family education and activation with continuing clinician education and preparation on medication deprescribing—a combination that has not been previously investigated.
- An effective pragmatic intervention to improve medication management for individuals with cognitive impairment can be taken to scale in multiple delivery systems and settings.

## Introduction

### Background and rationale {6a}

Most individuals with Alzheimer’s disease and related dementias (ADRD) or mild cognitive impairment (MCI) have multiple chronic conditions (MCC) [1]. The combination of cognitive impairment and chronic medical conditions leads to multiple medication use, complex medication regimens, and more potentially inappropriate medications (PIMs) [2]. Polypharmacy in individuals with cognitive impairment is associated with greater risk of adverse drug events and cognitive changes, and higher rates of hospitalization and mortality [3–9]. Optimizing medications through deprescribing can improve outcomes for MCC patients, particularly for those with ADRD or MCI [10–13]. Deprescribing, defined as the process of reducing or stopping the use of inappropriate medications or medications unlikely to be beneficial, can potentially benefit individuals with MCC and dementia or cognitive impairment.

Deprescribing interventions in ADRD populations have been largely limited to inpatient or skilled nursing settings or focused on specific classes of potentially inappropriate medication (e.g., anti-psychotics, statins) [14–18]. Evidence from the general older adult population suggests that interventions that are multidisciplinary, multifaceted, patient-centered, and provide “direct to consumer” information are the most effective at reducing inappropriate medication use [19–26]. There is a need to design and test interventions for the population with cognitive impairment that target multiple medications, can be integrated into regular practice, and are therefore sustainable.

Although physicians have expressed concern that patients and families may be resistant to deprescribing, over 90% of older individuals are receptive to discontinuing unnecessary medications when recommended by their physician [27–30]. Fostering patient and family interest in deprescribing may be key to its implementation [31, 32]. Patient and family education is most effective when coupled with activation achieved through considering meaningful questions around specific topics [33–36]. Engagement of primary care clinicians providers contributes to success [26]. Clinician behavior change is more likely to succeed through multidimensional interventions with interactive educational components, on topics relevant to current practice that are implemented on different levels [37, 38]. Although limited in number, interventions targeting more than one class of medications have also been successful [39]. The current intervention incorporates these essential features and examines the effectiveness of an intervention that couples patient and family education and activation with clinician preparation—a combination that has not been previously investigated with respect to deprescribing [40, 41].

## Methods/Design

### Study aims and objectives {7}

The study objective is to conduct and test a pragmatic, primary care-based, deprescribing intervention to educate and engage patients, family members, and primary care clinicians about deprescribing as one potential element of optimal medication management for patients with cognitive impairment and MCC. The intervention targets older adults with either dementia or mild cognitive impairment plus at least one other chronic condition who are taking 5 or more chronic medications. The primary outcomes are number of chronic medications and number of PIMs. Secondary outcomes include the number of PIM starts, dose reductions for selected PIMs (benzodiazepines, opiates, and antipsychotics), rates of adverse drug events (falls, hemorrhagic events, and hypoglycemic events), ability to perform activities of daily living, and skilled nursing facility, hospital, and emergency department admissions.

**Fig. 1:**
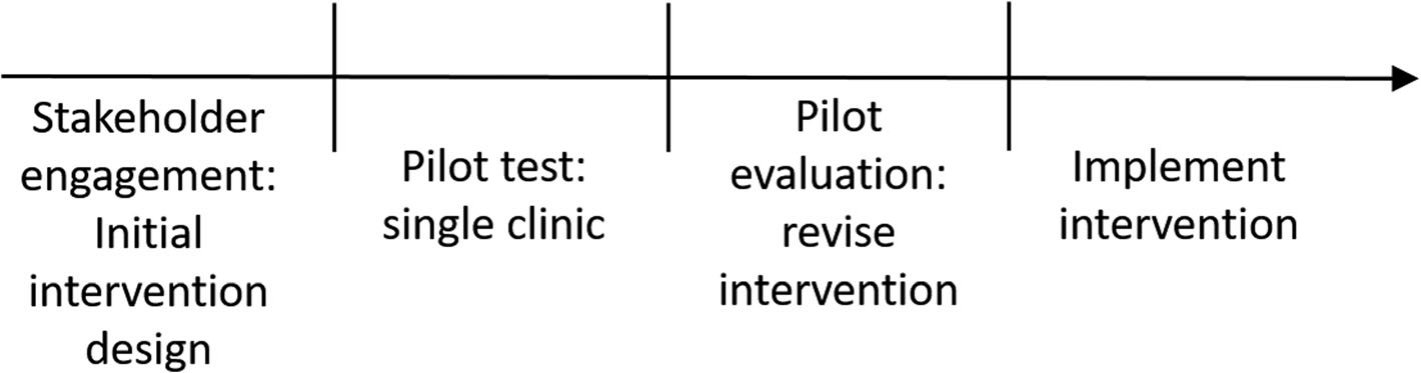
Activity sequence for intervention development, pilot testing, and implementation

The patient- and family-centered intervention was designed with extensive stakeholder engagement and piloted to ensure acceptability and feasibility. Below, we briefly describe initial intervention development and pilot evaluation of that initial intervention. We then provide details of the resulting full intervention (including content of the intervention materials) and the full intervention protocol. Figure 1 illustrates the sequence of intervention development, pilot testing, and implementation.

### Stakeholder engagement for intervention development

The intervention was conceptualized as having two components: a patient/ family component focused on education and activation about the potential value of deprescribing, and a clinician component focused on increasing clinician awareness about options and processes for deprescribing. It is based in part on the Chronic Care Model, in which an informed, empowered patient and family has productive interactions with a prepared, proactive practice team to improve outcomes [33, 42]. Input on intervention design and materials was sought from interviews with patients, family members, primary care clinicians, a patient/family advisory panel, and a clinician advisory panel. Primary care clinicians included physicians (the majority), mid-level advanced practice providers (such as Nurse Practitioners or Physician Assistants), and clinical pharmacists. Patient and family interviews focused on perceptions of deprescribing, desired information for considering deprescribing, and “talk back” reflections on study materials. Interviews with primary care clinicians explored facilitators and barriers to deprescribing, knowledge gaps, and approaches to communicating with patients and families.

### Pilot evaluation

The intervention, as initially designed, was evaluated in a 4-month pilot evaluation which included further stakeholder feedback. The objective of the pilot evaluation was to assess intervention feasibility and acceptability and to refine the intervention as needed to achieve these outcomes. Feasibility was assessed by successful completion of intervention tasks (identify and reach the target population, provide clinician materials, measure outcomes), and acceptability was assessed qualitatively through debriefing interviews with patients/family members and a primary care clinician focus group.

The pilot evaluation was conducted at a single primary care office at Kaiser Permanente Colorado (KPCO). The clinic had 12 physicians and 3 advanced practice providers caring for adult patients in primary care; 131 patients met eligibility criteria (age ≥ 65, dementia or MCI plus at least one additional chronic condition, and ≥ 5 chronic medications). Eligible patients and family members were mailed initial intervention materials before primary care visits and clinicians received periodic educational materials about potential approaches to deprescribing discussions in diverse clinical situations. We conducted 4 debriefing interviews with patients and family members and a focus group with clinicians at the pilot site to assess intervention acceptability. Interview and focus group questions focused on comprehension, awareness, ease of use, and relevance of intervention materials as well as the processes for distributing materials.

#### Pilot evaluation results and intervention refinement

Patients and family members found the intervention materials and processes to be acceptable and were open to deprescribing conversations although many did not realize that medication discontinuation could be a routine part of care. They recommended materials be sent twice over 12 months (as opposed to just once as in the pilot). Clinicians found the materials useful as they were open to deprescribing but were often unaware of how to initiate discussions. They requested additional “pearls” for deprescribing conversations that could be framed around common clinical scenarios, such as risk of medication side effects or discussions on goals of care. They also requested materials be distributed in hard copy at monthly meetings and that they receive a notification on their electronic schedule about mailings prior to upcoming patient visits.

To assess the feasibility of extracting primary and secondary outcomes from clinical data at scale, we developed a system-wide historical cohort of hypothetically eligible patients. All data-based outcomes were extractable from the electronic health record (EHR) or the KPCO Virtual Data Warehouse (VDW)—a quality-controlled common data model derived from multiple KPCO data sources (EHR, membership, pharmacy, laboratory, and other clinical data) [43]. Following the pilot evaluation, we expanded the study population to include individuals with MCI in addition to those with dementia. This eligibility change was based on the potential for individuals with MCI to benefit from deprescribing and widespread under-coding of dementia in most care delivery settings [44].

### Protocol for the OPTIMIZE trial

#### Overview of design {8}

**Fig. 2:**
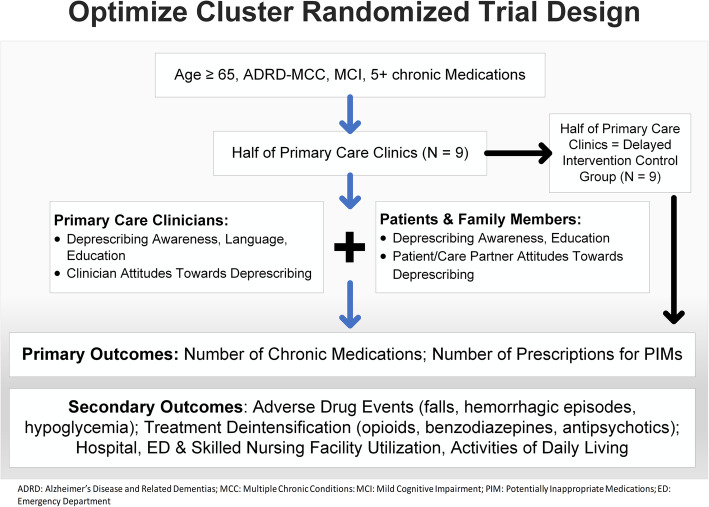
Study design for the OPTIMIZE pragmatic cluster randomized deprescribing intervention

The intervention is a pragmatic, cluster (*n* = 18 clinics) randomized trial to educate and activate individuals with ADRD or MCI and MCC and polypharmacy, their family members and their primary care clinicians about options and processes for deprescribing, with the goals of decreasing the number of chronic medications and number of PIMs and improving clinical outcomes. It is delivered at the clinic level with a wait-list control design. As a pragmatic intervention, it is designed to be straightforward, have broad inclusion/exclusion criteria, and be implemented across the KPCO system. The intervention has two components: a patient/family component focused on education and activation about deprescribing and a clinician component focused on increasing awareness among primary care providers about options and processes for deprescribing in the ADRD or MCI and MCC population (Fig. 2).

#### Study setting and intervention period {9} {6b}

The intervention is being conducted in Kaiser Permanente Colorado (KPCO), an integrated, not-for-profit healthcare delivery system that provides healthcare to more than 628,000 members throughout Colorado, including over 75,000 patients over 65. The patient population of KPCO is demographically representative of Colorado. The 18 primary care clinics in the Denver-Boulder service delivery area have been randomized into intervention sites (*n* = 9) and delayed intervention sites (i.e., control, *n* = 9) in blocks of two based on the number of patients assigned to primary care providers in each clinic (the primary care clinic that was used for the pilot evaluation has been excluded from further involvement in this study). The initial intervention period at each site will run for 12 months (March/April 2019 to February/March 2020), followed by a 12-month observational phase. The delayed intervention period (for control sites) will run for 12 months (approximately March 2021 to February 2022).

#### Study Population {10}

##### Patient inclusion and exclusion criteria

Age ≥ 65, having a primary care clinician in the KPCO Denver-Boulder service area, diagnosis of ADRD or MCI from ICD-9 or ICD-10 visit codes or from the problem list in the EHR, one or more additional chronic conditions (from a list 86 chronic medical conditions) [45], and taking 5 or more chronic medications. Of this eligible population, those who have at least one appointment with a primary care clinician which was scheduled at least 7 days in advance during the intervention period will receive the patient portion of the intervention. Given patient and clinic scheduling patterns, we anticipate that the intervention will reach 65% of the eligible population during a 12-month period. As the intervention is based in primary care clinics, individuals residing in long-term care facilities or enrolled in hospice care at baseline are excluded.

##### Clinician inclusion and exclusion criteria

Primary care clinicians who care for adult patients (including family medicine and internal medicine physicians and advanced practice providers) in the Denver-Boulder service area are included in this study. While the primary target of the clinician intervention is physicians (as patients select physicians as primary care continuity providers), other clinical staff (for example, advanced practice nurses) will receive clinician intervention materials if they attend primary care clinic provider meetings. They may also care for intervention patients during the intervention period.

##### Family member participation

Family members or other care partners are often involved in the care of individuals with cognitive impairment. Family members of patients meeting eligibility criteria may review and respond to intervention materials and accompany patients to medical appointments. Intervention materials encourage patients to share information with relevant family members.

### Recruitment {15}

Eligible patients are identified by study personnel using EHR and VDW data every weekday during the intervention period. Those with upcoming appointments within 7 to 14 days as indicated in the electronic schedules for the intervention clinics are mailed study materials and are entered into a study database for tracking. Patients at control sites who would be considered eligible for mailings receive usual care and are also identified and tracked for outcome measurement.

### The intervention {11a} {11d}

*The patient (and family) intervention* consists of materials mailed to all individuals meeting eligibility criteria at intervention clinics. The materials consist of (1) an informational brochure introducing the topic of discontinuing unnecessary or PIMs as part of optimal medication management and (2) the 9-question, validated revised Patients’ Attitudes Towards Deprescribing, version for cognitive impairment (rPATDcog or “Patients’ Attitudes”) questionnaire which captures the beliefs, attitudes, and experiences of people about deprescribing [31, 46–51]. The informational brochure is titled “Managing Medication, Could you benefit from taking fewer medicines?” and includes mention of how to plan for a visit to discuss medication reduction and reasons why some people may benefit from taking fewer medicines. It encourages interested patients and family members to discuss any interest in deprescribing with their primary care clinician at a primary care visit. The brochure specifically includes instructions NOT to discontinue any medications without talking to their primary care clinician. The mailing includes a stamped self-addressed envelope to return the questionnaire to the research team. Completing the Patients’ Attitudes questionnaire is intended to engage and activate patients and family members to consider deprescribing as part of optimal medication management and to increase awareness of deprescribing. While the Patients’ Attitudes questionnaire could be useful within the patient-clinician interaction, this was not a component of our intervention and the responses are not provided to primary care clinicians [34]. A cover letter provides general information about the study including encouragement for patients to share materials with family members plus contact information for the KPCO principal investigator and project manager. Patients receive materials within the 2-week period prior to a scheduled visit with their primary care clinicians. Mailings are repeated for up to 2 primary care clinicians’ appointments at least 2 months apart during the intervention period and include a second questionnaire if one has not been previously returned.

*The clinician intervention* consists of three elements. First, an initial 15- to 20-min educational presentation at the clinic department monthly provider meeting focuses on deprescribing as an element of optimal medication management for older adults with ADRD. During the presentation, providers are asked to complete the 9-question Prescribers’ Perceptions of Medication Discontinuation (PPMD) assessment [52, 53]. The validated PPMD instrument contains two domains (“patients’ clinical characteristics” and “clinicians’ perceptions of patients’ future health”) predictive of clinicians’ comfort with making discontinuation decisions. As with the Patients’ Attitudes questionnaire, answering the PPMD questions is intended to activate primary care clinicians to consider deprescribing as part of high-quality care. The second element is 12 one-page (single sided) “Tip Sheets” (see Table 1) on deprescribing that are distributed at monthly provider meetings for 12 months. Tip Sheets are sent by internal mail from the research team to the clinic staff member responsible for the agenda for the monthly meetings. The Tip Sheets, developed based on clinician input during the pilot study, contain suggested approaches and language to use in specific medication discontinuation situations.

**Table 1:** Tip Sheet topics for OPTIMIZE clinician intervention

1	Clinician guidance for deprescribing – an overview
2	Introduce deprescribing to patients
3	Deprescribing to improve troubling symptoms
4	Prescribing is a discussion opportunity
5	Recognize prescribing cascades
6	Reduce burden through deprescribing
7	Consider treatment deintensification
8	Discontinue risky medications to avoid adverse outcomes
9	Deprescribing as patients approach the end of life
10	Discuss deprescribing with family and friends
11	Don't forget about over the counter products
12	Summary document: deprescribing poster listing Tip Sheet topics

**Fig. 3:**
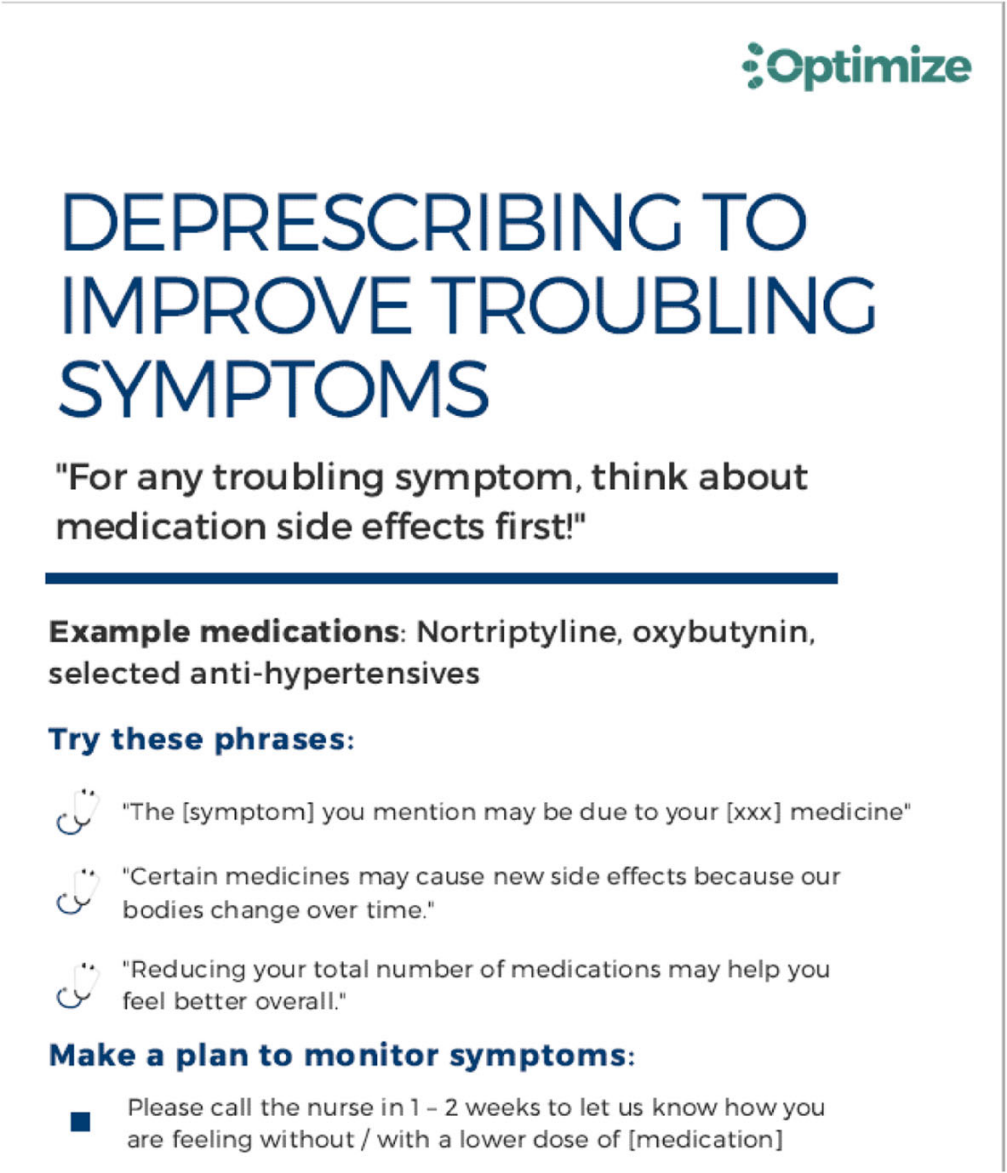
Example monthly deprescribing Tip Sheet for clinicians

For example, one Tip Sheet addresses deprescribing medications commonly used for symptom management that may increase the risk of falls or other adverse events, and another Tip Sheet addresses deprescribing medications unlikely to benefit individuals with limited life expectancy. Figure 3 provides an example of a monthly Tip Sheet. The third clinician intervention element is a one-sentence notification in the electronic appointment schedule that the patient has been mailed a patient intervention brochure.

### Control group {6b}

As a pragmatic trial randomized at the clinic level, 9 primary care clinics in the KPCO Denver-Boulder service delivery area serve as control sites. Eligible patients at these sites receive usual care throughout the initial intervention period. Patients and clinicians at control clinics are not intentionally blinded but are unaware of the intervention. Individuals meeting eligibility criteria at the control clinics are identified daily by study staff and entered into a database for tracking outcomes. Clinicians at the control sites do not receive any additional training or materials on deprescribing. Control clinics will receive the intervention in a delayed manner over a 12-month period starting 24 months after the start of the intervention at the other 9 clinics. This design will enable all eligible KPCO members and primary care clinicians to receive education about enhanced medication management through deprescribing.

### Treatment assignment procedures {16} {17}

Clinics are randomized in blocks of two by the study biostatistician, based on the number of patients assigned to primary care providers in each clinic. This blocking scheme ensures comparable numbers of eligible patients between intervention and delayed control groups. Larger clinics also have operational differences, such as separate internal medicine departments instead of combined primary care (family medicine and internal medicine combined) which are also aligned by the blocking scheme. After randomizing clinic assignments, we confirmed comparability of eligible members on age, gender, proportion on 5+ medications, and proportion on PIMs between the intervention and delayed control clinics. Limited remaining differences will be described and adjusted for in statistical analyses (see Table 2).

**Table 2:** Characteristics of intervention and control participants based on cohort used for sample size estimation^a^

	Total *N* = 3671	Intervention *N* = 1814 (49.4%)	Delayed control *N* = 1857 (50.6%)	Intervention vs delayed control *p* value^b^
1+ Potentially inappropriate Medication *N* (%)	1226 (33.4%)	598 (33.0%)	628 (33.8%)	0.58
Age in years, mean (SD)	79.6 (7.4)	79.6 (7.5)	79.6 (7.3)	0.83
Female gender	2047 (55.8%)	1025 (56.5%)	1022 (55.0%)	0.37
Non-white race^b^*N* (%)	444 (13.3%)	247 (14.5%)	197 (12.0%)	0.03
Missing race *N* (%)	325 (8.9%)	110 (6.1%)	215 (11.6%)	< 0.001
Hispanic ethnicity *N* (%)	441 (12.2%)	128 (7.2%)	313 (17.1%)	< 0.001
Missing ethnicity *N* (%)	48 (1.3%)	25 (1.4%)	23 (1.2%)	0.71

Comparisons for *p* values included: female vs male gender; non-white race vs white, excluding missing race; missing race vs not missing; Hispanic ethnicity vs non-Hispanic, excluding missing ethnicity; missing ethnicity vs not missing

^a^As of November 2018. Excluding 5+ medications limitation, total cohort = 8183 (intervention = 4049; delayed control 4134)

^b^Chi-square *p* values except for age (*t* test *p* value)

### Outcome measures {12} {13}

**Fig. 4:**
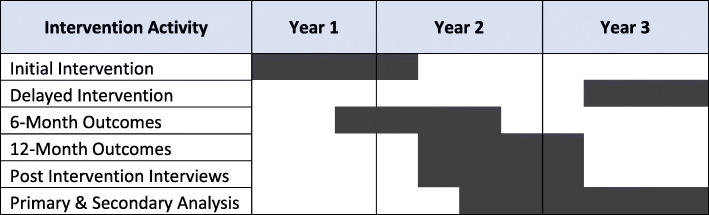
Timeline of intervention activities

Outcomes will be measured at 6 and 12 months after intervention completion (see Fig. 4). Primary outcomes are number of chronic medications and number of PIMs. Secondary outcomes include the number of PIM starts, dose reductions for selected PIMs (benzodiazepines, opiates, and antipsychotics), rates of adverse drug events (falls, hemorrhagic events, and hypoglycemic events), ability to perform activities of daily living, and skilled nursing facility, hospital, and emergency department admissions. Secondary outcomes will be measured in relevant subpopulations (e.g., dose reductions for individuals taking selected PIMs, hypoglycemic events for individuals with diabetes).

### Measure definitions and data sources {18a} {19}

Unless otherwise mentioned, all electronic data will be extracted from the KPCO VDW that includes data from multiple KPCO data sources covering the following domains: health plan enrollment, utilization, pharmacy, tumor registry, demographics, lab results, mortality, vital signs, census data, geocoded demographic data, patient reported outcomes collected in the course of care, problem list diagnoses, and social history [43].

#### Medications

Total number of chronic medications will be assessed for study eligibility and as a primary outcome at 6 and 12 months. Chronic medication use is defined as any prescription medication for which the patient had at least a 28-day supply on the assessment date. We selected a 28-day supply (rather than a supply for a longer time period) because some medications (such as opioid medications) are mostly dispensed in 28-day supplies. Chronic medications exclude the following domains identified by 2-digit GPI codes: vaccines, toxoids, allergenic extracts, oxytocics, local anesthetics—parenteral, general anesthetics, antiseptics and disinfectants, antidotes, diagnostic products, chemicals, and medical devices. Number of PIMs will also be assessed as 6- and 12-month outcomes, based on the well-established Beers list plus opioids [54] (see Additional file 1). Starts of PIMs (a secondary outcome) will be defined as new prescriptions in the absence of a prescription for that medication during the previous 6 months. Because calculating dose reductions requires analyzing specific medications, we will assess dose reductions in three selected drug classes: benzodiazepines, opiates, and antipsychotics. We selected these classes based on the premise that the medications posed risks of adverse side effects, but that patients, family members, and clinicians may be reluctant to fully discontinue the medications for fear of recurrent symptoms or physiologic withdrawal. As adverse drugs reactions are often dose related, dose reduction of these medications may still represent a meaningful outcome. We will examine dosages pre and post the intervention among persons on these medications in both time periods.

#### Diagnoses

Diagnosis codes are used to determine study eligibility and morbidity burden. ICD-9 (historical) and ICD-10 codes are used to identify diagnoses from visit billing codes, hospital and emergency department claims data, and problem list diagnoses and are measured during the 18 months prior to cohort eligibility determination. ICD9 and 10 codes for dementia and MCI used to assess study eligibility are listed in Additional file 2. Comorbid chronic diagnoses are from a list of 86 chronic conditions itemized in the Multiple Chronic Conditions Chartbook [45].

#### Adverse drug events (ADEs)

As secondary outcomes, we will examine three types of ADEs calculated from ICD codes that are common causes of emergency service use among older adults and that may decrease in a culture of thoughtful deprescribing: hypoglycemia in individuals with diabetes, falls, and hemorrhagic events. Denominators will use person-years to account for changes in enrollment and deaths.

#### Demographics

Demographic variables will be used to describe the cohort and as covariates in the analysis. Age, gender, and self-reported race/ethnicity will be extracted from the EHR. We will also assess census-based socioeconomic status and neighborhood deprivation index.

#### Utilization

Hospitalization rate, skilled nursing facility admission rate (temporary and permanent), and ED visit rate will be assessed as secondary outcomes.

#### Self-reported data

Ability to perform Activities of Daily Living will be assessed as a secondary outcome on the subset of patients who have a completed annual Medicare Health Risk Assessment which includes a list of Independent and Dependent Activities of Daily Living. An estimated at 45% of the KPCO Medicare beneficiary population have one or more completed Health Risk Assessments and these data are extractable from the EHR. Patient (or proxy) responses to the Patients’ Attitudes questionnaire completed and collected as part of the intervention will be incorporated into descriptive analyses. Results from the PPMD questionnaire collected during the clinician intervention will be used as a clinic level descriptor.

### Analytic plan {20a} {21b}

This study intervenes in two ways: activating physicians and educating patients on five or more medications with upcoming primary care appointments. To appropriately analyze impacts of these elements, we will utilize both cross-sectional and cohort analyses [55]. We will use cohort analyses to examine changes in medications among individuals eligible for brochure mailings. The cohort analysis start dates will vary by patient based on the first date of eligibility for a brochure mailing and will follow patients for 6 to 12 months. In addition to considering deprescribing medications for patients on five or more medications, physicians at intervention clinics may be less likely to initiate new medications. For this reason, the potential impacts of the physician component will be additionally analyzed by comparing medication counts overall and for PIMs at two points in time: the month prior to clinic study entry and 1 year after. For both cross-sectional and cohort analyses, we will compare the primary outcomes of counts of chronic medications and PIMs for intervention versus control groups using multilevel models to account for the clinic level randomization and clinic level intervention [56, 57]. The models will include random effects for clinic, for provider within clinic, and patient, and fixed effects for intervention versus delayed control and time (baseline or 1 year). These models can include baseline patient risk factors, as well as provider characteristics to account for imbalanced baseline covariates between clinics. We plan to use Poisson regression mixed models to examine change in the number of chronic medications and binomial regression mixed models for change in the proportion of patients on PIMs.

Multiple secondary outcomes will be examined. The following two examples illustrate planned analytic approaches to secondary outcomes: (1) Dose reductions, change over time: opioids are one specific drug category we will examine for prescribing patterns and dose reductions over time. We will identify persons on opioids at baseline and/or 1 year and compare proportions on opioids between intervention and control groups at the two time points using binomial regression models. We will examine dose reductions among the subgroup of persons on opioids at both time points by first converting the doses of varied drugs to Morphine Milligram Equivalents and then using linear mixed models to estimate change in mean doses between baseline and follow-up for intervention versus control clinics. (2) Rates of adverse outcomes among relevant subpopulations: we will examine rates of falls as a potential adverse drug event relevant for multiple medications that may decrease as a function of deprescribing. We will examine falls in the overall study population. Falls will be quantified using both ICD codes and, when available, patient- or proxy-reported fall data during the past 12 months using patient reported HRA data [58].

### Prespecified sub-analyses {20b}

We will examine effects of the intervention within three pre-planned subgroups [59]: (1) members who received a higher dose of the intervention (e.g., patient mailings before 2 primary care visits vs. before one visit during the intervention period, or those who have a visit with a provider but do not receive a brochure), (2) members on higher numbers of medications at baseline (7 or 8+ medications), and (3) individuals with MCI vs. ADRD. Within the intervention group, we will also compare respondents and non-respondents to the Patients’ Attitudes questionnaire at the individual level by demographic and morbidity characteristics. We will summarize and describe responses at the clinic level to the questionnaires used to engage and activate patients and clinicians as part of the intervention.

### Sample size {14}

There will be 9 intervention clinics and 9 control (delayed intervention clinics). We estimated power using members who would have been eligible for the intervention in November 2018. We identified 3671 eligible members with numbers per clinic ranging from 60 to 390 (Table 2). Current project estimates suggest that we will exceed this number and that approximately 4800 individuals from intervention or control clinics will contribute data to the analysis. For analyses of counts of medications, we will have > 80% power to detect an average decrease of − 0.70 (i.e., < 1 medication) based on differences of two Poisson rates in cluster-randomized design and assuming an event rate of 6.8 and intraclass correlation coefficient (ICC) = 0.01. We will be examining selected subgroups of interest, for example cohort members receiving a higher dose of the intervention or a restricted cohort with diagnosed dementia vs. MCI. Both of these comparisons could reasonably retain ~ 70% of the intervention cohort to contrast to the control cohort and we would be able to detect an average decrease of 1.4 medications even if ICC was doubled (i.e., 0.02). Comparisons of proportions of members on a PIM will be able to detect a decrease to 26.4% or less for the intervention clinics compared to the expected unchanged rate of 33.8% for the control clinics.

### Interim analyses and stopping rules {11b} {21b} {5d} {21a} {22} {25}

There are no prespecified statistical criteria that would suspend the intervention. Every 6 months, the Data Safety Monitoring Board (DSMB) reviews hospitalization and mortality rates (indicating potentially severe adverse drug withdrawal events) between groups who have received the intervention (have been sent a mailing) from intervention clinics, and those who would have been eligible to receive the initial mailing from control clinics (information on DSMB members and charter may be obtained from the National Institute on Aging). There is also ongoing safety monitoring using protocol-driven chart abstraction followed by blinded event adjudication to assess any potential clinical relationship between medication discontinuation and potential serious adverse events of hospitalization and mortality. All deaths and every third hospitalization occurring in the 4 months following brochure eligibility are reviewed [60]. Initial chart abstraction is conducted by PharmD chart abstractors who are not blinded to intervention status and assesses whether the event was preceded by primary care medication discontinuation or dose reduction. If medication discontinuation or dose reduction occurred in the primary care setting during that 4-month period, 3 clinically trained adjudicators who are blinded to intervention status independently evaluate whether the medication discontinuation or dose reductions possibly led or contributed to the event and whether the discontinuation/dose reduction was appropriate. In addition to adjudicators, study Principal Investigators are blinded to intervention status. Any serious adverse events potentially due to the intervention will be reviewed by the KPCO IRB and the DSMB.

### Human subjects protection {26a} {27} {30}

The study is approved by the KPCO Institutional Review Board (IRB) and all data are subject to confidentiality protection as Personal Health Information. Due to the pragmatic and educational nature of the intervention, the KPCO IRB has granted a waiver of informed consent for eligible patients and clinicians in the intervention clinics. The IRB approved providing an informational letter on the intervention as part of the patient mailing. The letter indicates that patients may wish to discuss medication discontinuation with their physician but are under no obligation to do so. Similarly, the clinician presentation delivered to intervention clinics includes a slide that provides general information on the study. Materials provided to clinicians and patients include contact information for the KPCO study Principal Investigator. Participation in the investigation does not affect KPCO members’ health insurance coverage.

## Discussion {31a}

This study will contribute evidence to inform achieving deprescribing as part of patient-centered care for older adults with ADRD or MCI plus MCC and polypharmacy in primary care. Unique features of this intervention include the simultaneous focus on patient and family within the context of primary care, the population at risk, and the pragmatic design. Planned mechanistic explorations include a qualitative investigation of intervention effectiveness and an assessment of physician decision making around deprescribing. Combined with the trial results, all these factors will inform dissemination as well as future implementation work on achieving deprescribing as a key part of regular primary care. Findings will be disseminated broadly to local stakeholders, and through national presentations and the peer-reviewed literature.

Study findings must be interpreted in context, understanding that the study setting is an integrated delivery system with good informational continuity on medication management. Although the setting could limit generalizability, integrated care models increasingly reflect other models for accountable care organizations or medical homes. Further, pragmatic interventions require integrated infrastructure to assess outcomes. We will not directly assess patient cognitive function pre- and post-intervention or self-report of health-related quality of life and patient or family experiences due to the pragmatic nature of the trial. However, we can assess patient and family members’ report of Activities of Daily Living from Medicare HRA data, providing insight into the functional impact of the intervention on individual patients. Additionally, we acknowledge that our primary outcome (chronic medication use) may not be considered, in isolation, to be a clinically or patient-important outcome. However, there is significant evidence of the harms of polypharmacy and the burden to individuals, their families, and the healthcare system. The objectives of this study are to achieve optimal appropriate medication use, through cessation and dose reduction of inappropriate medications, with a focus on safety and avoiding harms.

If the intervention is effective in reducing chronic medications, potentially inappropriate medications or in improving any of the secondary outcomes, it will demonstrate that educating patients, family members, and clinicians about deprescribing can contribute to high value care for older adults with cognitive impairment. If we do not see changes in outcomes, that implies that optimizing medication use through deprescribing may require a different time frame than this study and/or different approaches to preparing clinicians, patients, and families for deprescribing discussions for older adults with cognitive impairment.

### Trial status {2a} {2b} {3}

At the time of article submission, the intervention is being delivered to the initial intervention group under protocol Version 5, January 25, 2020. Recruitment began on March 6, 2019, and will be completed on approximately April 26, 2020. Clinicaltrials.gov registration number is NCT03984396.

### Adherence to reporting guidelines

Relevant protocol sections are mapped to SPIRIT checklist item numbers. In addition, the Consort 2012 checklist of information to include when reporting a cluster randomized trial is attached in Additional file 3.

## Supplementary information

**Additional file 1.** List of Potentially Inappropriate Medications (PIMS) used in outcome measurement.

**Additional file 2.** ICD 9 and ICD 10 codes used to identify patients with mild cognitive impairment and dementia.

**Additional file 3.** CONSORT 2012 checklist of information to include when reporting a cluster randomised trial.

## Data Availability

The datasets generated and/or analyzed during the current study are not publicly available as clinical data are the property of the healthcare delivery system and its patient members. Reasonable data requests will be considered by the authors with additional permission of the Kaiser Permanente health care system and its associated Institutional Review Board.
