# Supplementary material for: The OPTIMIZE patient- and family-centered, primary care-based deprescribing intervention for older adults with dementia or mild cognitive impairment and multiple chronic conditions: study protocol for a pragmatic cluster randomized controlled trial
Source: Trials. 2020 Jun 18;21:542. doi: 10.1186/s13063-020-04482-0 (PMC7301527; doi:10.1186/s13063-020-04482-0)
Supplement: Supplementary file 1 — Additional file 1. List of Potentially Inappropriate Medications (PIMS) used in outcome measurement. [file 13063_2020_4482_MOESM1_ESM.docx]

**Additional File 1.**

**List of Potentially Inappropriate Medications (PIMS) used in outcome measurement**

| **Category** | **Subcategory** | **Generic name** |
| --- | --- | --- |
| H2-receptor antagonists |  | Cimetidine |
|  |  | Famotidine |
|  |  | Nizatidine |
|  |  | Ranitidine |
| Anticholinergics  Anticholinergics | Antidepressants | Desipramine |
|  |  | Nortriptyline |
|  |  | Paroxetine |
|  |  | Amoxapine |
|  |  | Protriptyline |
|  |  | Trimipramine Maleate |
|  | Antiemetic | Prochlorperazine |
|  | Antihistamines | Meclizine |
|  |  | Brompheniramine |
|  |  | Carbinoxamine |
|  |  | Chlorphen-PE-PPA-Atropine |
|  |  | Chlorpheniramine |
|  |  | Clemastine |
|  |  | Cyproheptadine |
|  |  | Dexbrompheniramine |
|  |  | Dexchlorpheniramine |
|  |  | Diphenhydramine |
|  |  | Doxylamine |
|  |  | Hydroxyzine |
|  |  | Promethazine |
|  |  | Triprolidine |
|  | Antimuscarinics | Darifenacin |
|  |  | Fesoterodine |
|  |  | Oxybutynin |
|  |  | Solifenacin |
|  |  | Tolterodine |
|  |  | Trospium |
|  |  | Atropine |
|  |  | Dimenhydrinate |
|  |  | Flavoxate |
|  |  | Propantheline |
|  |  | Umeclidinium |
|  | Antispasmodics | Aclidinium Bromide |
|  |  | Clidinium and Chlordiapoxide |
|  |  | Dicyclomine |
|  |  | Homatropine |
|  |  | Hydrocodone with Homatropine |
|  |  | Hyoscyamine |
|  |  | Scopolamine |
|  |  | Belladonna Alkaloids |
|  |  | Difenoxin with Atropine |
|  |  | Diphenoxylate with Atropine |
|  |  | Methylatropine with Amylase |
|  | Other | Methscopolamine |
| Antipsychotics  Antipsychotics |  | Acepromazine |
|  |  | Aripiprazole |
|  |  | Asenapine |
|  |  | Brexpiprazole |
|  |  | Cariprazine |
|  |  | Chlorpromazine |
|  |  | Chlorprothixene |
|  |  | Clozapine |
|  |  | Droperidol |
|  |  | Fluphenazine Decanoate |
|  |  | Haloperidol |
|  |  | Iloperidone |
|  |  | Loxapine |
|  |  | Lurasidone |
|  |  | Mesoridazine Besylate |
|  |  | Molindone |
|  |  | Olanzapine |
|  |  | Paliperidone Palmitate |
|  |  | Perphenazine |
|  |  | Pimozide |
|  |  | Piperazine |
|  |  | Quetiapine |
|  |  | Reserpine |
|  |  | Risperidone |
|  |  | Thiethylperazine |
|  |  | Thioridazine |
|  |  | Trifluoperazine |
|  |  | Zipresidone |
| Benzodiazepine hypnotics |  | Estazolam |
|  |  | Flurazepam |
|  |  | Midazolam |
|  |  | Quazepam |
|  |  | Temazepam |
|  |  | Triazolam |
| Benzodiazepines |  | Alprazolam |
|  |  | Chlordiazepoxide |
|  |  | Clorazepate |
|  |  | Diazepam |
|  |  | Halazepam |
|  |  | Lorazepam |
|  |  | Oxazepam |
|  |  | Prazepam |
| Nonbenzodiazepine hypnotics |  | Ergoloid Mesylates |
|  |  | Eszopiclone |
|  |  | Isoxsuprine |
|  |  | Zaleplon |
|  |  | Zolpidem |
| Opioids |  | Codeine |
|  |  | Fentanyl |
|  |  | Hydrocodone |
|  |  | Hydromorphone |
|  |  | Methadone |
|  |  | Morphine |
|  |  | Oxycodone |

Reference:

American Geriatrics Society 2015 Updated Beers Criteria for Potentially Inappropriate Medication Use in

Older Adults. *J Am Geriatr Soc, 63*(11), 2227-2246.
