# Supplementary material for: The OPTIMIZE patient- and family-centered, primary care-based deprescribing intervention for older adults with dementia or mild cognitive impairment and multiple chronic conditions: study protocol for a pragmatic cluster randomized controlled trial
Source: Trials. 2020 Jun 18;21:542. doi: 10.1186/s13063-020-04482-0 (PMC7301527; doi:10.1186/s13063-020-04482-0)
Supplement: Supplementary file 2 — Additional file 2. ICD 9 and ICD 10 codes used to identify patients with mild cognitive impairment and dementia. [file 13063_2020_4482_MOESM2_ESM.docx]

**Additional File 2.**

**ICD-9 and ICD-10 codes for dementia and MCI used to assess study eligibility**

| **ICD** | **Code** | **Definition** |
| --- | --- | --- |
| 09 | 290 | Dementias |
| 09 | 290.0 | Senile dementia, uncomplicated |
| 09 | 290.1 | Presenile dementia |
| 09 | 290.10 | Presenile dementia, uncomplicated |
| 09 | 290.11 | Presenile dementia with delirium |
| 09 | 290.12 | Presenile dementia with delusional features |
| 09 | 290.13 | Presenile dementia with depressive features |
| 09 | 290.2 | Senile dementia with delusional or depressive features |
| 09 | 290.20 | Senile dementia with delusional features |
| 09 | 290.21 | Senile dementia with depressive features |
| 09 | 290.3 | Senile dementia with delirium |
| 09 | 290.4 | Vascular dementia |
| 09 | 290.40 | Vascular dementia, uncomplicated |
| 09 | 290.41 | Vascular dementia, with delirium |
| 09 | 290.42 | Vascular dementia, with delusions |
| 09 | 290.43 | Vascular dementia, with depressed mood |
| 09 | 290.8 | Other specified senile psychotic conditions |
| 09 | 290.9 | Unspecified senile psychotic condition |
| 09 | 294.0 | Amnestic disorder in conditions classified elsewhere |
| 09 | 294.1 | Dementia in conditions classified elsewhere |
| 09 | 294.10 | Dementia in conditions classified elsewhere without behavioral disturbance |
| 09 | 294.11 | Dementia in conditions classified elsewhere with behavioral disturbance |
| 09 | 294.2 | Dementia, unspecified, without behavioral disturbance |
| 09 | 294.21 | Dementia, unspecified, with behavioral disturbance |
| 09 | 294.8 | Other persistent mental disorders due to conditions classified elsewhere |
| 09 | 310.89 | Mild memory disturbances, not amounting to dementia, associated with senile brain disease |
| 09 | 331 | Other cerebral degenerations |
| 09 | 331.0 | Alzheimer’s disease |
| 09 | 331.1 | Frontotemporal dementia |
| 09 | 331.11 | Picks disease |
| 09 | 331.19 | Other frontotemporal dementia |
| 09 | 331.2 | Senile degeneration of brain |
| 09 | 331.8 | Other cerebral degeneration |
| 09 | 331.82 | Dementia with Lewy bodies |
| 09 | 331.83 | Mild cognitive impairment |
| 09 | 331.89 | Other cerebral degeneration |
| 09 | 331.9 | Cerebral degeneration, unspecified |
| 09 | 780.93 | Memory loss |
| 09 | 797 | Senility without psychosis |
| 09 | 799.52 | Cognitive communication deficit |
| 10 | F01.50 | Vascular dementia without behavioral disturbance |
| 10 | F01.51 | Vascular dementia with behavioral disturbance |
| 10 | F02.80 | Dementia in other diseases classified elsewhere without behavioral disturbance |
| 10 | F02.81 | Dementia in other diseases classified elsewhere with behavioral disturbance |
| 10 | F03.90 | Unspecified dementia without behavioral disturbance |
| 10 | F03.91 | Unspecified dementia with behavioral disturbance |
| 10 | F03.92 | Presenile with delusional features |
| 10 | F03.93 | Presenile with depressive features |
| 10 | F06.8 | Other specified mental disorders due to known physiological condition |
| 10 | F07.89 | Mild memory disturbances, not amounting to dementia, associated with senile brain disease |
| 10 | F19.97 | Other psychoactive substance use, unspecified with psychoactive substance-induced persisting dementia |
| 10 | G30.0 | Alzheimer’s disease with early onset |
| 10 | G30.1 | Alzheimer’s disease with late onset |
| 10 | G30.8 | Other Alzheimer’s disease |
| 10 | G30.9 | Alzheimer’s disease, unspecified |
| 10 | G31.01 | Picks disease |
| 10 | G31.09 | Other frontotemporal dementia |
| 10 | G31.1 | Senile degeneration of brain, not elsewhere classified |
| 10 | G31.83 | Dementia with Lewy bodies |
| 10 | G31.84 | Mild cognitive impairment |
| 10 | G31.89 | Other specified degenerative diseases of nervous system |
| 10 | G31.9 | Degenerative disease of nervous system, unspecified |
| 10 | R41.3 | Other amnesia |
| 10 | R41.81 | Age-related cognitive decline |
| 10 | R41.841 | Cognitive communication deficit |
